# Adaptation of a Standard Method for Water Absorption Testing of Stone Materials: The Case of a Hydrophilic Protective Coating

**DOI:** 10.3390/ma16124228

**Published:** 2023-06-07

**Authors:** Gabriel Búrdalo-Salcedo, Indira Rodríguez, María Fernández-Raga, Sagrario Fernández-Raga, Carlos Rodríguez-Fernández, José Miguel González-Domínguez

**Affiliations:** 1 Department of Chemistry and Applied Physics, Universidad de León, 24071 León, Spain; iroda@unileon.es; 2 Department of Architectural Theory and Architectural Design, Universidad de Valladolid, 47014 Valladolid, Spain; sagrario.fernandez@uva.es (S.F.-R.); carlos.rodriguez.fernandez@uva.es (C.R.-F.); 3 Carbon Nanostructures and Nanotechnology Group (G-CNN), Instituto de Carboquímica (ICB-CSIC), 50018 Zaragoza, Spain; jmgonzalez@icb.csic.es

**Keywords:** porosity, absorption, coatings, method, graphene oxide

## Abstract

The historical stone heritage that we inherit must be passed on to future generations, not only in the same conditions that we found it but, if possible, in better ones. Construction also demands better and more durable materials, often stone. The protection of these materials requires knowledge of the types of rocks and their physical properties. The characterization of these properties is often standardized to ensure the quality and reproducibility of the protocols. These must be approved by entities whose purpose is to improve the quality and competitiveness of companies and to protect the environment. Standardized water absorption tests could be envisaged to test the effectiveness of certain coatings in protecting natural stone against water penetration, but we found that some steps of these protocols neglect any surface modification of the stones, and hence may not be completely effective when a hydrophilic protective coating (i.e., graphene oxide) is present. In this work, we analyze the UNE 13755/2008 standard for water absorption and propose alternative steps to adapt the norm for use with coated stones. The properties of coated stones may invalidate the interpretation of the results if the standard protocol is applied as is, so here we pay special attention to the characteristics of the coating applied, the type of water used for the test, the materials used, and the intrinsic heterogeneity of the specimens.

## 1. Introduction

The great majority of the historic buildings constructed up to the second half of the 19th century were built using construction materials found in their immediate surroundings, optimizing the natural resources of their areas [1]. The selection of the different materials responded to mechanical quality, availability, and workability criteria. Mechanical quality is directly related to the porosity and mechanical strength of the different varieties available: high mechanical resistance can guarantee a resistant and durable construction, while low porosity can render difficult the cutting and extraction of blocks. At the end of the 19th century, a regulatory framework was created by the European Union to ensure that new constructions guaranteed structural stability.

The European Council Directive 89/106/EEC of 21 December 1988 on construction products—amended by Directive 93/68/EEC of 21 July 1993—established the essential requirements, conformity assessment systems, and CE marking for construction products. Since then, numerous harmonized standards have been published for natural stone products. Currently, any manufacturer must declare the physical–mechanical characteristics of the rocks they extract from their quarry as part of the product identification in both the declaration of performance and the CE marking.

Water absorption at atmospheric pressure is one of the most important specifications of rocks—together with density, hygrothermal properties, elastic properties, flexural strength, frost resistance, etc. Absorption values can be calculated following a standardized voluntary protocol (UNE-EN 1341, UNE-EN 1469, UNE-EN 12057, and UNE-EN 12058) [2,3,4,5]. The value of water absorption enables the evaluation of the stone’s liquid infiltration performance [6] and, consequently, of its ease of staining, alteration with chemical agents, and proliferation of micro-organisms.

The performance of the product is frequently measured by exposition to humidity, spills, or liquid splashes found in kitchens and bathrooms in restaurants, cafeterias, supermarkets, gymnasiums, etc. In general terms, the products requiring a special degree of protection and those requiring a special degree of sanitation—used in hospitals, kindergartens, etc.—should present the lowest possible absorption value. Water absorption can also represent a useful indicator of susceptibility to frost damage. The American Society for Testing Materials (ASTM) standards and the Building Technological Standard for Floor Covering: Rigid Pieces (NTE-RSR for the Spanish acronym) offer recommended values for absorption. Another aspect to be considered is the potentially different behavior of frequently used stones, protected with coatings to improve their resistance, and artificial stones, such as silestone or dekton.

In the present work, we analyze the most commonly used and recommended standard for the characterization of stone specimens: the UNE 13755/2008 [7] standard. After finding that the results of several water absorption tests on coated specimens yielded values that were impossible to interpret and even showed contradictory trends, we decided to check all the steps of the standard protocol. A series of deficiencies were detected in this standard protocol when the specimen to be tested presented some kind of surface coating. The main deficiencies found are listed below:

● It does not consider the presence of coatings.
● It does not consider the temperature at which the coatings are no longer stable.
● It does not specify the composition of the water to be used.
● It does not specify the type of cloth to be used to collect the water from the test specimens after immersion in water.
● It does not consider the inherent heterogeneity of the materials to be tested.

Given that these standards represent a benchmark for a product or service quality tested under specific conditions, we consider it to be of the utmost importance to analyze certain aspects, and the objective of this work is to provide a series of guidelines for work with natural stones presenting some type of coating.

## 2. Materials and Methods

### 2.1. Materials and Equipment

**Figure 1:**
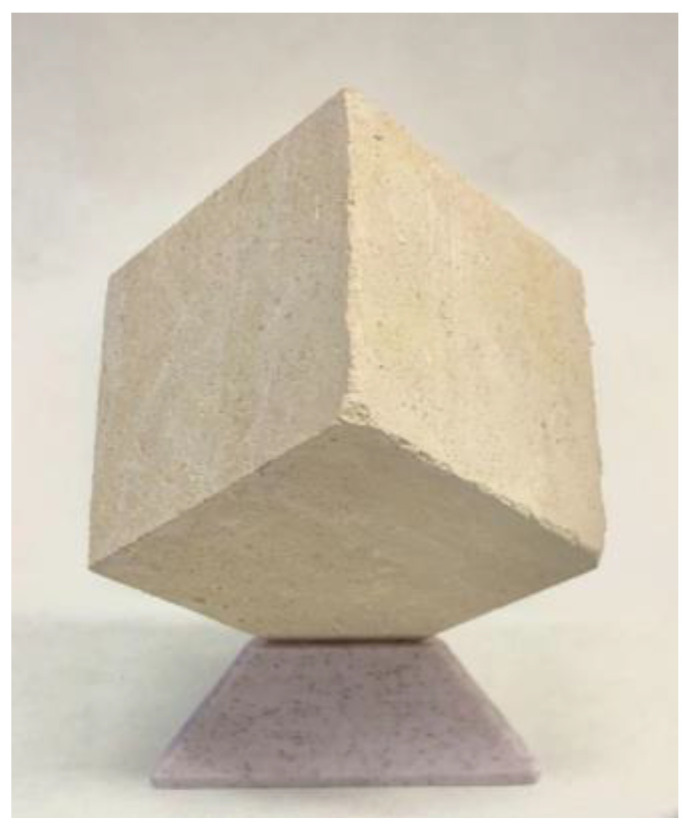
Boñar (Spain) limestone specimen as delivered from quarry (50 mm edge cube).

In the present study, we used cubic stone specimens of 50 ± 5 mm of edge (Figure 1). The type of rock used in this work can be classified as dolomite—known as limestone, from Boñar, Spain—a fine-grained, crystalline, and highly dolomitized rock [8,9,10]. It presents sparitic limestone filling in fissures and cavities. The average porosity of this type of rock is 5%, and the bulk density is 2.78 g/m^3^ [11]. We chose this kind of stone specimen as it has been widely used in numerous buildings; its physical properties—despite its easy workability—are prone to degradation, which significantly influences the effect of water on pore wall behavior.

We used common labware to perform the water absorption test: a caliper with a 0.01 g accuracy, a ventilated oven, and a desiccator. To immerse the specimens in water, we used a flat-bottomed polyethylene container with a nonabsorbent stainless-steel holder.

### 2.2. Methods

The methodology followed in the present work was based on the one used in the UNE 13755/2008 standard with modifications responding to the application of this standard to a material presenting a protective coating. We quantified the contribution of each modification to the final result.

Each test, based on the standard and its modifications, was performed on a specific number of specimens divided into three groups: specimens in natural state, specimens superficially coated with a concentration of 6.6 μg/cm^2^ of graphene oxide (GO), and specimens superficially coated with a concentration of 9.9 μg/cm^2^ of GO. These values have been determined as the optimal surface concentrations of a protective coating in previous studies [12].

#### 2.2.1. UNE-EN 13755/2008 Standard

UNE-EN 13755 is currently the only standard specifying a method for determining the water absorption of natural stone by immersion in water at atmospheric pressure. Its principle is to dry the specimens to a constant mass and then immerse them in water at atmospheric pressure for a specific period of time until a constant mass is achieved. The water absorption—expressed as a percentage—is calculated as the ratio between the difference in mass between a saturated specimen and a dry specimen and the mass of the dry specimen, as expressed in Equation (1):

(1) $$A_{b}=\frac{\left(m_{s}-m_{d}\right)}{m_{d}}\times 100$$

where *A_b_* is absorption, *m_s_* is the saturated specimen mass, and *m_d_* is the dry specimen mass. This equation expresses the percentage of water that can be absorbed by the specimen.

According to the standard, specimens must present a cylinder, cube, or prism shape of 70 ± 5 mm or 50 ± 5 mm side/diameter and must be obtained by diamond-saw coring or cutting. Their apparent volume—calculated from their geometric measurements—must be at least 60 mL. In addition, the ratio between surface area and volume must be between 0.08 mm^−1^ and 0.20 mm^−1^. The minimum number of specimens indicated for this test is 6.

The specimens must be dried previous to the test at a temperature of 70 ± 5 °C until a constant mass is obtained. This mass is reached when the difference between two weights measured consecutively at an interval of 24 ± 2 h is not more than 0.1% of the mass of the first measurement. To weigh the specimens, they must have reached room temperature; to achieve this state, specimens are placed in a desiccator following their removal from the oven until a temperature of 20 ± 5 °C is reached. The result of the last stabilized weighing is the mass of the dry specimen (*m_d_*).

Once the specimens have lost all the water content in their pores, the actual absorption test can begin. As stipulated in the standard, specimens must be placed in a flat-bottomed container. Each specimen must be at least 15 mm from adjacent specimens or from the vessel wall. This vessel should be filled with running water at 20 °C up to half the height of the specimens. These conditions represent the initial time t_0_. After 1 h (t_0_ + 60 ± 5 min), additional water is poured in to cover 3/4 of the height of the specimens; 1 h later (t_0_ + 120 ± 5), additional water is poured in until a height of 25 ± 5 mm above the top surface of the specimens is reached.

In the period of time from that moment up until t_0_ + 48 ± 2 h, specimens are removed from the water and dried superficially using a damp cloth to remove the excess surface water. The time spent for the surface drying and subsequent weighing of each specimen is about 30 s. Specimens are then again immersed in water and the test continues. Every 24 ± 2 h, specimens are removed from the water, dried, and weighed following the procedure described above. As mentioned above, constant mass is reached when the difference between two weights measured consecutively does not exceed 0.1% of the mass of the first measurement. The result from the last stabilized weighing is the mass of the saturated specimen (*m_s_*).

With the values for the masses of the dry and saturated specimens, the absorption value can be calculated using Equation (1).

#### 2.2.2. UNE-EN 13755/2008 Standard Limitations

The European standard UNE-EN 13755/2008—“Natural stone test methods—Determination of water absorption at atmospheric pressure”—specifies the methodology to be applied only to natural stone specimens for the determination of water absorption, but does not consider the possibility of these specimens presenting some kind of chemical and/or physical surface coating. To date, no applicable standard considering this aspect can be found in the literature.

**Figure 2:**
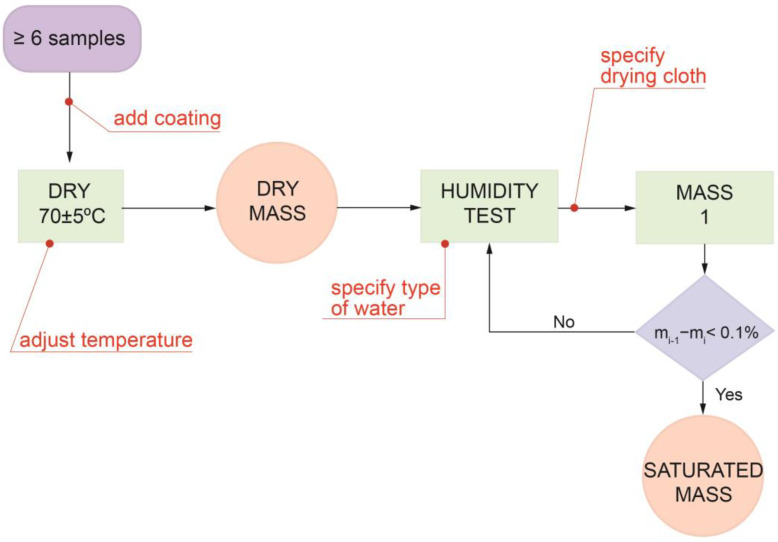
Flow diagram of the process followed to obtain the dry mass (*m_d_*) and the saturated mass (*m_s_*) necessary to calculate the porosity according to the UNE-EN 13755/2008 standard. In red, the points where intervention is needed to improve this standard.

The potential effects of a protective coating on natural stone are varied: waterproofing, color changing, protection from micro-organisms, avoiding water accumulation in its interior, etc. These treatments can affect the mechanical behavior of the rock, and therefore the tests performed to evaluate this characteristic must be adapted to the type of coating. We used GO [12]; this hydrophilic coating creates a surface film around the dolomite cubes, and therefore it is important to distinguish the water retained on the surface (i.e., GO network) from the water inside the stone. In the present study, we designed and followed a new protocol able to consider the hydrophilic protection of natural stone specimens. Our protocol is based on the UNE-EN 13755/2008 standard with the addition of the analysis of the following aspects (Figure 2):

- Type of coating and its influence on water or stone;
- Temperature and pressure conditions;
- Suitability of all the materials used during the test;
- Influence of the water used on the rock or on the coating.

##### Protective Surface Coatings

**Figure 3:**
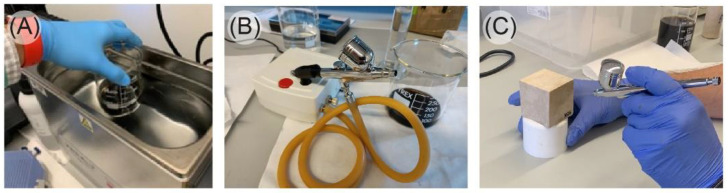
Specimen preparation: (**A**) ultrasonic-bath-based dispersion of GO, (**B**) airbrush connected to the pump, and (**C**) airbrush application.

Natural stone specimens that need to be tested can present coatings such as varnishes, damp-proofing materials, ceramic nanoballs, or nanocoatings such as StoLotusan paint or siloxane paint [13]. In this work, we used GO as coating due to its recently demonstrated excellent protective efficacy on dolostone [12]. This product is in a powder state; however, we used colloidal dispersion for its application as this allows for the use of an airbrush. GO must be dispersed before its application on the stones. GO is easily dispersed in water by ultrasound or mechanical agitation [14]. For the present work, we chose a 2 min ultrasonic bath, as GO is highly hydrophilic and does not require a great amount of energy for dispersion and 2 min is enough time to ensure good dispersion without damaging the molecular structure of the carbon [15,16]. Subsequently, we used an airbrush to spray the colloidal dispersion of GO in water on the specimens (Figure 3). The specimens received different GO surface concentrations, namely 6.6 µg/cm^2^ and 9.9 µg/cm^2^.

After application of the coating, 24 h had to elapse before the specimen could be tested. As per protocol, the dry specimen was placed in an oven at 70 ± 5 °C for a certain period of time to measure its mass, which could then be compared with the mass of the saturated specimen. However, while GO at room temperature is metastable [17], it starts to decompose at temperatures between 50 °C and 70 °C [18,19], with accelerated decomposition occurring at temperatures above 100 °C [20]. Therefore, we adapted the procedure to avoid loss of the coating protective properties, and unacceptable darkening (undesirable for ornamental stones) occurred upon thermal reduction of GO.

The coated specimens were placed in an oven at 30 °C for 5 days to reach the *m_s_*. The resulting mass was the same as that obtained by weighing the dry specimens without any coating, which were subjected to 70 °C heat to reach *m_d_*.

##### Excess Water Removal after Specimen Immersion

For the standard, the use of a damp cloth ensures that the water removed from the specimen when taken out of the water represents excess water solely from the surface in the form of droplets, and the interior of the shallower pores does not lose water content, thus avoiding the underestimation of *m_s_*.

**Figure 4:**
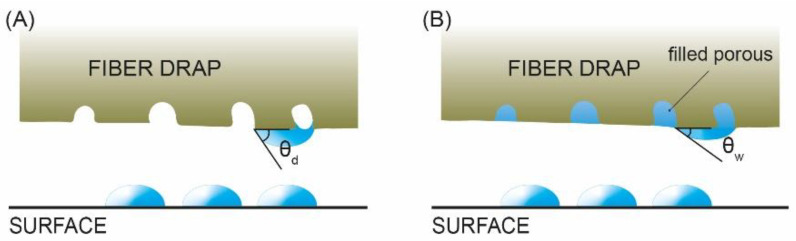
Schematic drawing of the contact angle on a dry cloth surface (**A**) and on a damp cloth surface (**B**). Modified from [22].

Many wipes or drapes are made of a material called viscose rayon: a semisynthetic fiber made from natural sources of regenerated cellulose [21]. In this type of fabric, it has been proven that the contact angle of dry rayon is 30–40°, which is high enough to prevent absorption. Upon interaction with water, many hydrogen bonds in the cellulose fibers are formed, causing the exposed hydroxyl groups to drag a large amount of water molecules that remain attached, swelling the fibers. Free hydroxyl groups interact with water on the surface, and the contact angle consequently drops because the absorption of water into a cellulosic tissue occurs mainly by capillarity, which requires a low meniscus contact angle [22], resulting in water absorption (Figure 4).

The use of other types of cloths has been found to leave residues on the test specimens, thus modifying the surface chemical composition when incinerated during the heat treatment.

We used surgical drapes in the present work to avoid contamination of the specimens and ensure that only surface water was removed from the surface.

##### Water Composition during Absorption Tests

The main alterations caused by water on stone materials are due to the action of dissolved substances of different gaseous species, such as CO_2_. The transport of ions, such as Cl^−^, SO_4_^2−^, Na^+^, K^+^, Ca^2+^, Mg^2+^, etc., can cause the precipitation of salts inside the porous system of rocks, with its consequent disruptive effect. In the case of water saturation, this can lead to an increase in volume of those component materials containing swellable minerals (clays) or of the fluid itself, due to the effect of freeze–thaw cycles [23,24]. In addition, soluble salts within the voids can significantly increase and occasionally decrease the damage due to frozen water. In terms of mechanical properties, a loss of strength is observed as the degree of water saturation of a rock increases [25]. The current standard protocol does not specify which type of water the specimens should be immersed in. Tap water contains chemical elements in quantities that must not exceed certain limits imposed by the Ministry of Health [26].

In the specific case of calcareous rock, the salts dissolved in water could react with the composition of calcite, its major component. Calcite is soluble in water under certain conditions, such as the presence of ammonium salts and carbon dioxide (Equation (2)) [27]. Tap water contains sodium, potassium, calcium, magnesium, chlorine, sulfur, and phosphorus to make it drinkable [28]; chlorine can react with calcium (Equation (3)).

CaCO_3_ + H_2_O → Ca^2+^ + 2OH^−^ + CO_2_ (2)

CaCO_3_ + 2HCl → CaCl_2_ + H_2_O + CO_2_ (3)

**Figure 5:**
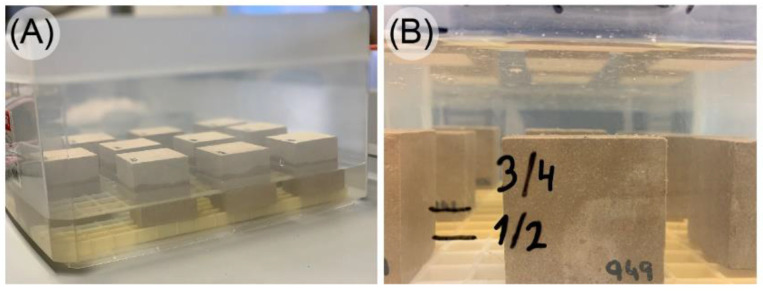
Specimen immersion steps during the absorption test: (**A**) in the first step, water covers half the height of the specimens and (**B**) in the last step, water surface reaches 2.5 cm above the top surface of the specimens.

Working with distilled or deionized water is considered more appropriate. Therefore, in the present work, we used distilled water to immerse the specimens in (Figure 5).

##### Overcoming the Native Heterogeneity of Stones

A rock, and more so a sedimentary rock, naturally presents heterogeneities in its composition, fabric, and texture that cannot be controlled. Even rocks from the same quarry can present different geomechanical properties, depending on the position of the layer from which these were extracted, and exhibit different behavior [29,30]. The physical properties of different rock types are determined by their composition, their formation mechanism, and the external processes that may have affected them. The pores are distributed as a three-dimensionally interconnected network, even establishing nodes where different conduits converge, making evident the capacity of the rock to retain or store a fluid (liquid or gas) in its bulk, which has repercussions in the elastic and mechanical behavior and in chemical phenomena [25,31]. That is why one of the most interesting properties from a civil engineering point of view is porosity; this is difficult to measure as it strongly varies from specimen to specimen, and the classical method to assess it (i.e., mercury porosimeter) could be considered destructive because Hg irreversibly seeps through the stone pores and cannot be recovered. To overcome the problem of heterogeneity, when calculating the absorption percentage of a stone specimen and comparing it with the absorption percentage after coating application, we performed measurements for all tests on the exact same specimens. That is, after testing a natural specimen, it was brought back to its dry mass, GO coatings were applied on it, and the test was performed again. This can be repeated several times if the coating is transpirable.

## 3. Results

### 3.1. UNE-EN 13755/2008 Standard

**Figure 6:**
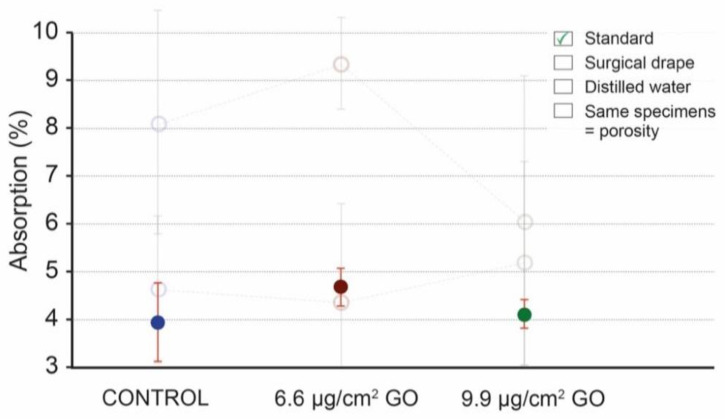
Water absorption mean values of control specimens (blue), specimens coated with 6.6 μg/cm^2^ of GO (red), and specimens coated with 9.9 μg/cm^2^ of GO (green); mean values (dots) and standard deviation (vertical lines), following the standard. The empty dots and lines with transparency correspond to previous tests following the standard and the filled dots with total opacity correspond to the last test which has been used in this work to compare the data. It is possible to appreciate that the trend in the three tests is contradictory.

Figure 6 presents the values obtained by applying the specifications of the standard protocol (Appendix A: Table A1). The results of the water absorption calculation after the humidity test according to the standard specifications are 3.94% for the control (uncoated) specimens and 4.68% and 4.11% for the specimens coated with 6.6 μg/cm^2^ of GO and specimens coated with 9.9 μg/cm^2^ of GO, respectively [12]. The standard deviation (SD) in each group shows low values, between 0.29 and 0.81.

### 3.2. Use of Surgical Drape to Collect Excess Water after Specimen Immersion

**Figure 7:**
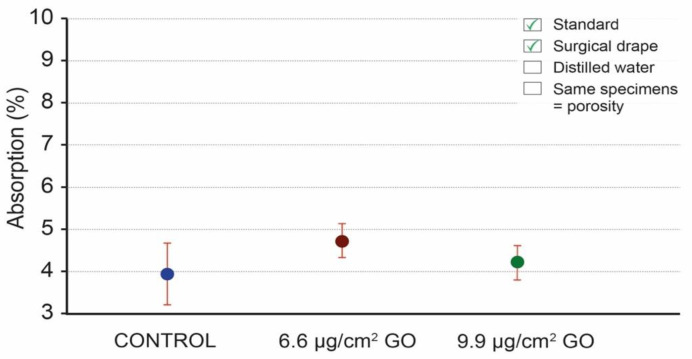
Water absorption mean values of control specimens, specimens coated with 6.6 μg/cm^2^ of GO, and specimens coated with 9.9 μg/cm^2^ of GO; mean values (dots) and standard deviation (red lines), using surgical drapes while removing the excess water after the immersion step.

The first protocol modification implemented to improve the critical points of the standard was to use a surgical drape to remove the excess water from the specimens after being immersed in water for a more accurate weighing (Video S1). This surgical drape was selected because it represents a clean and cheap alternative that, in addition, does not leave any residue on the specimens that could be deposited or even burnt during the oven-drying step, contaminating the specimen and modifying its weight. Figure 7 presents the absorption values obtained using this method (Appendix A: Table A2). The mean values of absorption using surgical drapes to remove the excess water after the immersion step are 3.92% for the control specimens and 4.71% and 4.19% for the specimens coated with 6.6 μg/cm^2^ of GO, and specimens coated with 9.9 μg/cm^2^ of GO. The SD in each group ranges between 0.41 and 0.79.

### 3.3. Use of Distilled or Deionized Water

**Figure 8:**
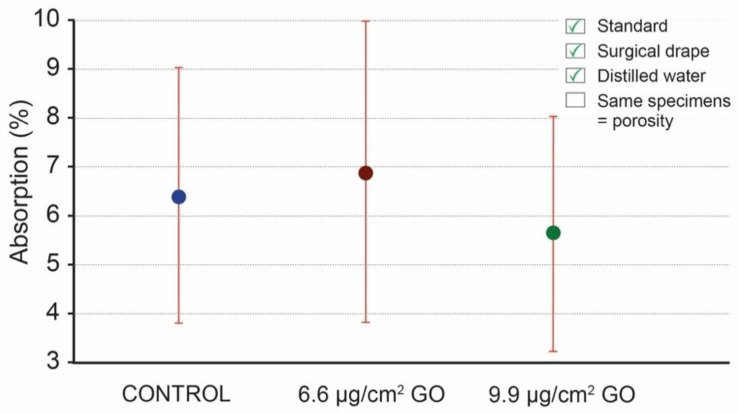
Water absorption mean values of control specimens, specimens coated with 6.6 μg/cm^2^ of GO, and specimens coated with 9.9 μg/cm^2^ of GO; mean values (dots) and standard deviation (red lines), using surgical drapes and distilled water.

The second protocol modification implemented was the use of distilled water for the immersion of the specimens. Figure 8 presents the absorption values obtained using this type of water (Appendix A: Table A3). The mean values of absorption are 6.38% for the control specimens and 6.86% and 5.62% for the specimens coated with 6.6 μg/cm^2^ of GO and specimens coated with 9.9 μg/cm^2^ of GO, respectively. The SD in each group shows high values, between 2.41 and 3.09.

### 3.4. Use of the Same Specimens for Testing to Overcome the Heterogeneity Problem

**Figure 9:**
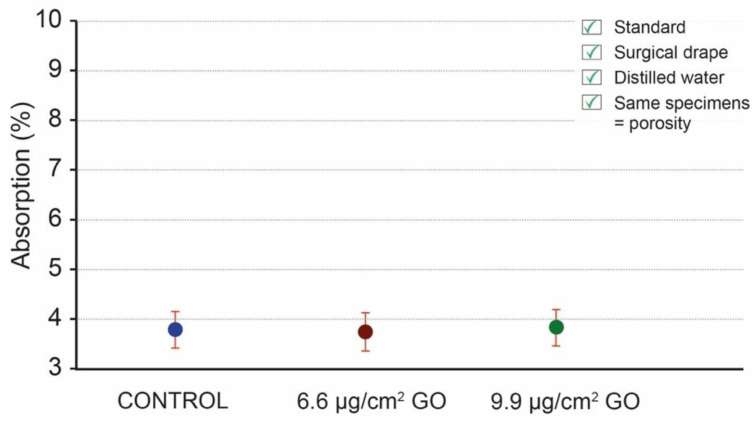
Water absorption mean values of control specimens, specimens coated with 6.6 μg/cm^2^ of GO and specimens coated with 9.9 μg/cm^2^ of GO; mean values (dots) and standard deviation (red lines), using the same specimens.

The third protocol modification implemented was the use of the exact same specimens for the control and the overlay tests. Figure 9 presents the porosity values obtained using this method (Appendix A: Table A4). The mean values of porosity are 3.78% for the control specimens and 3.75% and 3.84% for the specimens coated with 6.6 μg/cm^2^ of GO and specimens coated with 9.9 μg/cm^2^ of GO, respectively. The SD in each group shows significantly low values, between 0.36 and 0.37.

**Figure 10:**
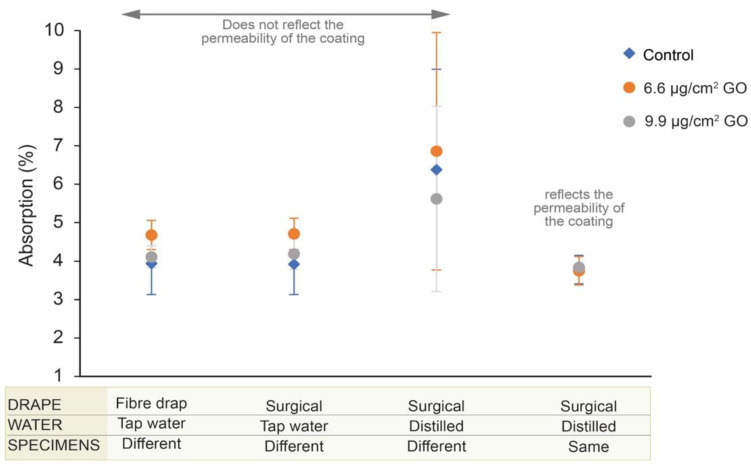
Summary of all tests specifying the modifications and indicating which values are more accurate. The graph shows the water absorption mean values of control specimens, specimens coated with 6.6 μg/cm^2^ of GO, and specimens coated with 9.9 μg/cm^2^ of GO; mean values (dots) and standard deviation (vertical lines).

The variability in the values for each test and their standard deviations can be clearly seen in Figure 10. The results of the test in which all modifications have been made show the most similar values and the smallest standard deviation.

## 4. Discussion

The most common pathologies present in stone materials are derived from the action of external agents, such as physicochemical attack from atmospheric agents and the substances transported by them, from the proliferation of organisms and microorganisms, and from ultraviolet rays, marine aerosols, etc. [32,33,34,35,36]. These pathologies can affect both the aesthetics of the rock and its mechanical behavior, as physical or chemical attacks alter its petrophysical properties. These aspects are important in the restoration field and, above all, for cultural heritage.

Given that, in many cases, alteration processes depend on the circulation of liquids inside porous solids, the behavior of the materials with respect to water is crucial to avoid the degradation associated with the retention of water inside the voids in rocks, such as pores or fractures; circumstances of cyclical freezing and thawing; the crystallization of soluble salts, e.g., saline efflorescence, subflorescence, etc.; and in the case of the transport of harmful substances [37].

Since porosity (and therefore the percentage of water absorption) is one of the most significant features, especially when referring to industrial rocks, the standardized procedures to measure it must consider a series of critical aspects that can determine the outcome. Our absorption calculation on cubic specimens of calcareous stone coated with a protective material revealed certain shortcomings in the standardized protocols.

The protective nanomaterial we chose for the present study was GO, due to its demonstrated efficacy in the field of stone surface protection and in cultural heritage [12,38]. This coating creates a hydrophilic surface protection which, in turn, acts as a molecular sieve for water when in the form of droplets, as in the case of torrential rain simulations [12,39]. However, this coating is not impermeable when the specimens are completely submerged in water, as seen in this study, since the coating allows for the moisture balance to be maintained. Therefore, if the coated specimens are immersed in water, the water will eventually fill the pores. After performing an absorption test following the UNE-EN 13755/2008 standard, we found that the results obtained did not reflect the permeability of GO in specimens submerged in water. The values of 3.94% for control specimens and more than 4.1% for coated specimens seemed to indicate that the GO allowed for higher adsorption or increased porosity. Absorption was higher even with a lower protection of 6.6 μg/cm^2^ of GO (4.68%) than with a protection of 9.9 μg/cm^2^ of GO (4.11%; Table A4). These results seemed contradictory, as a higher surface concentration of such an impermeable coating should entail lower (or at least unchanged) water absorption. Having performed the three tests in the same conditions and obtaining different and unrelated trends (Figure 6), we sought the possible cause within the protocol itself.

Figure 10 shows how the obtained results vary to a greater or lesser extent with each protocol modification, until reasonable values are reached. As can be seen in Table A2 and Table A3 (Appendix A), the values from the water absorption tests (control, 6.6 μg/cm^2^ of GO, and 9.9 μg/cm^2^ of GO) differ greatly. The use of a surgical drape to collect the excess water from the specimens before measuring the *m_s_* resulted in water absorption values similar to those obtained in the standard (unmodified) test. The use of distilled water to avoid a possible interference of ions with the carbonates of the stones resulted in a considerable increase in the absorption values, presenting percentages higher than 5.6%. The minimum value was obtained in the specimens coated with 9.9 μg/cm^2^ of GO, contrary to the previous tests. The use of the exact same specimens for all tests resulted in values (Appendix A: Table A3) that were similar among themselves (SD: 0.36–0.37). The average absorption for the uncoated specimens was 3.78%, a coating of 6.6 μg/cm^2^ of GO resulted in an average of 3.75%, and a coating of 9.9 μg/cm^2^ of GO resulted in an average of 3.84%. These values show the critical influence of the heterogeneity of the stone specimens currently used in the physical characterization tests. These results indicate that rock heterogeneity must be considered when comparing tests. The repetition of tests with and without different coatings on the same samples is advisable.

The dolomites from Boñar, Spain, are sedimentary rocks that, due to their origin—and the physical and chemical processes that occurred during their formation—present a greater textural heterogeneity than that of metamorphic or igneous rocks. These are subjected to such high pressures and/or temperatures that their minerals unite, resulting in a more homogeneous texture. For studies testing metamorphic or igneous rocks—such as shales or granites—the last step proposed in our protocol can therefore be omitted—provided that homogeneity is analyzed. Despite the seemingly contradictory results, we consider our first two protocol modifications appropriate whenever working with coated stones. All the protocol modifications implemented in the present study should be considered whenever heterogeneous rocks are used and/or protective coatings are applied.

As mentioned above, in the present work, we applied a hydrophilic carbon-based nanomaterial (GO) on the surface of calcareous stones. For this type of coating in particular, one of the most important control points is the maximum permissible temperature for drying the specimens. Besides the values of the absorption test, the color of the specimens can change when subjected to temperatures above a threshold value. GO is a coating material used on historical heritage stones and should not alter the aesthetics of the buildings on which it is applied, hence the importance of the potential color change. Not only the stability conditions of the coating but also the coating application method used and the type of rock tested should be considered.

## 5. Conclusions

The interaction with water triggers or accelerates stone decomposition through supervening situations such as washing by rain, freezing and thawing, the migration and crystallization of soluble salts, chemical and biological attacks, the corrosion of metallic elements and wind erosion, high consumption of thermal energy, etc.

The protection of stone materials from the effects of water is advisable, although the protections applied must never obstruct the passage of the water vapor present on the walls or the capillarity water; the application of a protective product must ensure breathability of the rocks, so that the hygrometric values of the structures are kept constant, avoiding the dangerous internal stagnation of water. Traditional protocols to measure water absorption do not consider the presence of coatings such as GO and must therefore be adapted to this reality. In the present study, we followed a standardized protocol for water absorption testing and provide certain guidelines to adapt this protocol to the work with natural stones presenting a hydrophilic protective coating. The aspects we propose to consider in an alternative methodology based on the UNE-EN 13755/2008 standard are as follows:

● A specification of the presence of coatings and their quantity (preferably in terms of surface concentration). These may exert physical or chemical properties on stone surfaces that vary when subjected to the test conditions.
● The temperature at which the coatings are no longer stable. A stipulated temperature for drying the specimens and obtaining the weight of the dry specimen must be below the stability conditions of the coating, if present; otherwise, the test must be modified to ensure a valid result.
● The composition of the water to be used. We propose to prioritize the use of distilled or deionized water throughout the test, since tap water contains some ions which may react with the rock materials or the coating.
● The type of cloth to be used to collect the excess water from the test specimens after immersion in water. We propose the use of surgical drapes to collect the excess water present as droplets on the surface of the specimen when it is removed from the water to avoid contamination of the specimen.
● The heterogeneity of the material to be tested. We recommend to use the same specimens for all comparative analyses if possible when working with natural rocks, since the inherent heterogeneity of certain materials can be an important issue when interpreting the results obtained from the tests. Another compatible possibility could be to increase the number of specimens tested, consequently increasing the variation.

Any failure to consider these aspects may induce erroneous results in the absorption test, leading to invalid interpretations of the results.

## Data Availability

Details are not added.

## Supplementary Materials

The following supporting information can be downloaded at https://www.mdpi.com/article/10.3390/ma16124228/s1, Video S1: Use of surgical drape to remove the excess water after the immersion step.

## Appendix A

**Table A1:** Water absorption values of control specimens, specimens coated with 6.6 μg/cm^2^ GO, and specimens coated with 9.9 μg/cm^2^ GO; mean values and standard deviation (SD) following the standard. *m_d_*: dry mass; m_1_: first weighing after 48 h; m_2_: second weighing after 72 h; *m_s_*: saturated mass; *A*: absorption percentage; *A_mean_*: average absorption values; and sd: standard deviation.

	Sample Code	m_*d*_ (g)	m_1_ (g)	m_2_ (g)	m_*s*_ (g)	A (%)	A_*mean*_ (%)	sd
CONTROL (uncoated)	A1	303.80	316.60	316.90	316.90	4.31	3.94	0.81
	A2	268.80	281.70	281.90	281.90	4.87		
	A3	271.40	284.80	284.90	284.90	4.97		
	A4	287.80	298.90	299.20	299.20	3.96		
	A5	296.10	305.90	306.10	306.10	3.38		
	A6	311.40	320.80	321.10	321.10	3.11		
	A7	311.30	320.30	320.60	320.60	2.99		
6.6 μg/cm^2^ GO surface concentration	A8	289.30	300.40	300.70	300.70	3.94	4.68	0.38
	A9	265.50	278.90	279.10	279.10	5.12		
	A10	281.20	294.90	295.20	295.20	4.98		
	A11	270.30	283.10	283.40	283.40	4.85		
	A12	269.70	281.80	282.10	282.10	4.60		
	A13	276.60	288.90	289.20	289.20	4.56		
	A14	279.80	292.70	293.00	293.00	4.72		
9.9 μg/cm^2^ GO surface concentration	A14	309.10	320.70	321.00	321.00	3.85	4.11	0.29
	A16	282.40	294.40	294.60	294.60	4.32		
	A17	278.90	291.10	291.40	291.40	4.48		
	A18	293.20	306.00	306.20	306.20	4.43		
	A19	302.50	313.70	314.00	314.00	3.80		
	A20	286.40	297.50	297.80	297.80	3.98		
	A21	300.40	311.90	312.20	312.20	3.93		

**Table A2:** Absorption values of control specimens, specimens coated with 6.6 μg/cm^2^ GO, and specimens coated with 9.9 μg/cm^2^ GO; mean values and SD using surgical drapes while removing the excess water after the immersion step. *m_d_*: dry mass; m_1_: first weighing after 48 h; m_2_: second weighing after 72 h; *m_s_*: saturated mass; *A*: absorption percentage; *A_mean_*: average absorption values; and sd: standard deviation.

	Sample Code	*m_d_* (g)	m_1_ (g)	m_2_ (g)	*m_s_* (g)	*A* (%)	*A_mean_* (%)	sd
CONTROL (uncoated)	B1	304.25	316.46	316.86	316.86	4.14	3.92	0.79
	B2	269.10	281.69	282.06	282.06	4.82		
	B3	271.70	284.92	285.20	285.20	4.97		
	B4	288.16	299.24	299.61	299.61	3.97		
	B5	296.50	306.43	306.75	306.75	3.46		
	B6	311.90	321.41	321.65	321.65	3.13		
	B7	311.86	320.89	321.08	321.08	2.96		
6.6 μg/cm^2^ GO surface concentration	B8	289.76	300.78	301.15	301.15	3.93	4.71	0.41
	B9	265.87	279.43	279.65	279.65	5.18		
	B10	281.66	294.94	295.95	295.95	5.07		
	B11	270.69	283.23	283.58	283.58	4.76		
	B12	270.02	282.37	282.62	282.62	4.67		
	B13	276.91	289.53	289.61	289.61	4.59		
	B14	280.20	293.30	293.55	293.55	4.76		
9.9 μg/cm^2^ GO surface concentration	B15	309.48	321.28	321.61	321.61	3.92	4.19	0.41
	B16	282.77	294.43	294.86	294.86	4.28		
	B17	270.09	282.95	283.50	283.50	4.97		
	B18	293.59	306.10	306.59	306.59	4.43		
	B19	303.00	314.08	314.37	314.37	3.75		
	B20	286.84	297.91	298.26	298.26	3.98		
	B21	300.85	312.42	312.83	312.83	3.98		

**Table A3:** Absorption values of control specimens, specimens coated with 6.6 μg/cm^2^ GO, and specimens coated with 9.9 μg/cm^2^ GO; mean values and SD using surgical drapes and distilled water. *m_d_*: dry mass; m_1_: first weighing after 48 h; m_2_: second weighing after 72 h; *m_s_*: saturated mass; *A*: absorption percentage; *A_mean_*: average absorption values; and sd: standard deviation.

	Sample Code	*m_d_* (g)	*m_d_* (g) + GO	m_1_ (g)	m_2_ (g)	*m_s_* (g)	*A* (%)	*A_mean_* (%)	sd
CONTROL (uncoated)	C1	284.17	284.40	297.30	297.47	297.47	4.60	6.38	2.61
	C2	277.72	277.99	294.96	295.18	295.18	6.18		
	C3	298.54	298.71	307.79	307.96	307.96	3.10		
	C4	264.59	264.81	291.95	293.39	293.39	10.80		
	C5	286.08	286.26	305.69	306.71	306.71	7.15		
	C6	297.60	297.80	315.89	317.00	317.00	6.45		
6.6 μg/cm^2^ GO surface concentration	C7	302.42	302.61	312.86	312.99	312.99	3.43	6.86	3.09
	C8	319.98	320.14	329.32	329.54	329.54	2.94		
	C9	280.05	280.34	296.96	297.17	299.17	6.72		
	C10	264.88	265.06	289.21	290.82	290.82	9.72		
	C11	285.97	286.16	308.52	309.82	309.82	8.27		
	C12	272.24	272.44	298.03	299.92	299.92	10.09		
9.9 μg/cm^2^ GO surface concentration	C13	304.30	304.62	323.06	323.32	323.32	6.14	5.62	2.41
	C14	310.42	310.62	319.07	319.20	319.20	2.76		
	C15	270.15	270.49	288.25	288.53	288.53	6.67		
	C16	277.73	277.92	301.93	303.46	303.46	9.19		
	C17	306.99	307.16	324.52	325.44	325.44	5.95		
	C18	338.39	338.53	348.14	348.77	348.77	3.02		

**Table A4:** Absorption values of control specimens, specimens coated with 6.6 μg/cm^2^ GO, and specimens coated with 9.9 μg/cm^2^ of GO; mean values and SD using the same specimen for all three tests. *m_d_*: dry mass; m_1_: first weighing after 48 h; m_2_: second weighing after 72 h; *m_s_*: saturated mass; *A*: absorption percentage; *A_mean_*: average absorption values; and sd: standard deviation.

	Sample Code	*m_d_* (g)	*m_d_* (g) + GO	m_1_ (g)	m_2_ (g)	*m_s_* (g)	*A* (%)	*A_mean_* (%)	sd
CONTROL (uncoated)	D1	320.62	320.63	330.74	330.97	330.97	3.22	3.78	0.37
	D2	298.59	298.96	310.03	310.18	310.18	3.75		
	D3	332.21	332.58	344.83	345.07	345.07	3.76		
	D4	291.67	291.97	303.20	303.40	303.40	3.91		
	D5	317.51	317.88	331.27	331.58	331.58	4.31		
	D6	306.09	306.49	315.85	316.22	316.22	3.17		
	D7	326.94	327.27	339.04	339.27	339.27	3.67		
	D8	299.22	299.53	311.51	311.80	311.80	4.10		
	D9	300.08	300.38	311.20	311.53	311.53	3.71		
	D10	320.28	320.63	333.77	334.00	334.00	4.17		
6.6 μg/cm^2^ GO surface concentration	D1	320.58	320.82	330.75	330.85	330.85	3.13	3.75	0.37
	D2	298.52	298.79	309.92	310.00	310.00	3.75		
	D3	332.16	332.44	344.59	344.65	344.65	3.67		
	D4	291.64	291.88	303.18	303.26	303.26	3.90		
	D5	317.48	317.74	331.30	331.40	331.40	4.30		
	D6	305.98	306.28	315.97	316.22	316.22	3.25		
	D7	326.91	327.17	339.01	339.10	339.10	3.65		
	D8	299.19	299.41	311.38	311.50	311.50	4.04		
	D9	300.03	300.27	311.19	311.28	311.28	3.67		
	D10	320.26	320.52	333.59	333.70	333.70	4.11		
9.9 μg/cm^2^ GO surface concentration	D1	320.56	320.82	330.73	331.58	331.58	3.35	3.84	0.36
	D2	298.51	298.79	310.00	310.29	310.29	3.85		
	D3	332.14	332.41	344.60	344.85	344.85	3.74		
	D4	291.61	291.58	303.19	303.42	303.42	4.06		
	D5	317.45	317.73	331.28	331.65	331.65	4.38		
	D6	305.92	306.23	315.99	316.16	316.16	3.24		
	D7	326.91	327.20	339.03	339.40	339.40	3.73		
	D8	299.18	299.42	311.50	311.58	311.58	4.06		
	D9	300.03	300.24	311.18	311.53	311.53	3.76		
	D10	320.25	320.51	333.79	333.97	333.97	4.20		
